# Ga-In Alloy Segregation within a Porous Glass as Studied by SANS

**DOI:** 10.3390/nano13081357

**Published:** 2023-04-13

**Authors:** Andrei V. Uskov, Elena V. Charnaya, Aleksandr I. Kuklin, Min Kai Lee, Lieh-Jeng Chang, Yurii A. Kumzerov, Aleksandr V. Fokin

**Affiliations:** 1 Physics Department, St. Petersburg State University, 198504 St. Petersburg, Russia; a.uskov@spbu.ru; 2 Joint Institute for Nuclear Research, 141980 Dubna, Russia; kuklin@nf.jinr.ru; 3 Moscow Institute of Physics and Technology, 117303 Moscow, Russia; 4 Instrument Center of Ministry of Science and Technology at National Cheng Kung University, Tainan 70101, Taiwan; mklee@mail.ncku.edu.tw; 5 Department of Physics, National Cheng Kung University, Tainan 70101, Taiwan; ljchang@mail.ncku.edu.tw; 6 Ioffe Institute, 194021 St. Petersburg, Russia; yu.kumzerov@mail.ioffe.ru (Y.A.K.); midbarzin@yandex.ru (A.V.F.)

**Keywords:** silica porous glass nanotemplate, Ga-In alloy, SANS, simulation

## Abstract

Nanolattices can play the role of templates for metals and metallic alloys to produce functional nanocomposites with particular properties affected by nanoconfinement. To imitate the impact of nanoconfinement on the structure of solid eutectic alloys, we filled porous silica glasses with the Ga-In alloy, which is widely used in applications. Small-angle neutron scattering was observed for two nanocomposites, which comprised alloys of close compositions. The results obtained were treated using different approaches: the common Guinier and extended Guinier models, the recently suggested computer simulation method based on the initial formulae for neutron scattering, and ordinary estimates of the scattering hump positions. All of the approaches predicted a similar structure of the confined eutectic alloy. The formation of ellipsoid-like indium-rich segregates was demonstrated.

## 1. Introduction

Nanolattices can be used as templates for a variety of liquids and solids to produce new functional nanocomposites, whose properties are essentially driven by embedded substances. This offers absolutely novel opportunities for applications of nanolattices with filled inter-units space in different areas of nanoelectronics, chemistry, and medicine. Metals and metallic alloys of a low melting point are the most promising candidates to be introduced into the nanolattice inner space. The Ga-In alloy is widespread in traditional fields of electronics, techniques, and engineering, but it also has great perspectives in modern cutting-edge applications such as self-healing superconducting wires, contacts for sensors in wearable electronics and for soft robotics, thermal interface materials for components of micro- and nanoelectronics, and others [1,2,3,4,5,6,7]. Along with good thermodynamic features, the advantages of the Ga-In alloy include low toxicity, and high and stable electric conductivity.

The Ga-In alloy has a simple phase diagram of the eutectic type. The bulk Ga-In alloy segregates at freezing into two crystalline phases: an indium-rich solid solution with symmetry of bulk indium and an almost pure gallium phase with a very small amount of dissolved indium [8]. The stable gallium-rich phase has a symmetry of α-Ga. The segregates can be of different shapes, chiefly, lamellae and rods. The shapes and sizes of the segregates change following the eutectic alloy composition, cooling rate, and pressure. The sizes of segregates usually vary from several microns to a few hundred microns.

We should expect that nanostructuring of the Ga-In eutectic alloy, as a result of embedding it into rigid templates, deeply affects the phase diagram, as was found for some other eutectic alloys [3]. For instance, it was shown that the solidus and liquidus temperatures diminished and the mutual solubility ranges for solid solutions altered in the particular binary eutectic alloy nanostructures [9,10,11,12]. The structure of segregates can be also influenced by size effects as was demonstrated for the Ge-Sn nanoparticles which solidified under specific conditions into an amorphous phase in contrast to the bulk alloy [13]. Recent studies of superconductivity in some gallium-based eutectic alloys confined to nanoporous templates revealed strong changes in the temperature of the superconducting transitions [14,15,16].

The impact of nanoconfinement on the properties of the Ga-In alloy was studied by ultrasonic techniques and nuclear magnetic resonance (NMR) [17,18,19,20,21]. These experimental methods provided information on shifts in the solidus and liquidus lines, slow-down of atomic mobility in the melts, stabilization in the β-Ga structure in the gallium-rich segregates, and the emergence of the liquid–liquid transition in the supercooled nanoalloy. Nevertheless, the sizes and shapes of the segregates in the frozen Ga-In alloy under nanoconfinement remained poorly understood, as well as the geometry of segregates in other eutectic alloys embedded into nanopores.

Recently, we showed that small-angle neutron scattering (SANS) proved to be quite efficient in studying the size and shape of segregates in the solid Ga-In nanoalloy confined within a synthetic opal [22]. The purpose of the present work consists in the application of SANS to estimate the geometric parameters of the Ga-In segregates within a porous silica glass template. We will use four different approaches to treat experimental SANS data: the common Guinier and extended Guinier models developed for independent scatters of arbitrary shapes [23,24], the computer simulation method based on the initial formulae for neutron scattering suggested in [22], and ordinary estimates for the wave vector at the scattering hump. We will show that the approaches predict a consistent image of the confined alloy structure.

## 2. Materials and Methods

**Figure 1:**
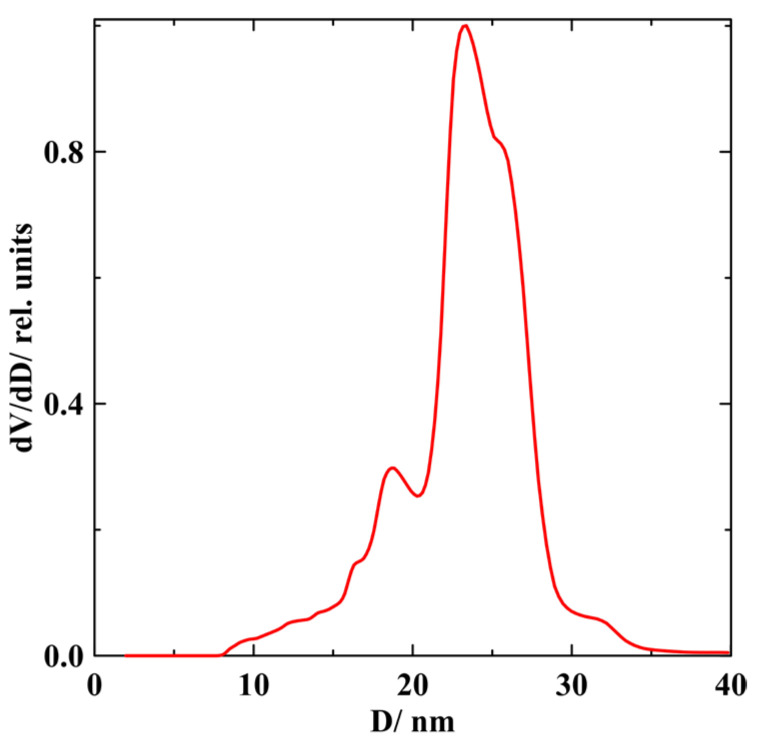
The normalized pore volume distribution dV/dD (*D* is the pore diameter) in the porous glass template.

The porous silica template was made of a phase-separated sodium borosilicate glass through acid leaching, which produced the interconnected network of pores. The mean pore size and size distribution were obtained with nitrogen adsorption–desorption porosimetry using Quadrasorb SI. Pore size distribution in the silica glass template is shown in Figure 1. The mean pore diameter is equal to 23 nm. The total pore volume was evaluated by weighing the porous glass before and after filling it with decane and was found to be 18%. The melted Ga-In alloy of two compositions was embedded into pores under pressure of up to 10 kbar. The compositions of these alloys were 94 at.% Ga/6 at.% In and 96 at.% Ga/4 at.% In. The 1.5 mm thick plates for SANS studies were cut from the filled porous glasses. The plate surfaces were cleaned from bulk alloys.

The scattering intensity dependence on wave vector was measured at the YuMO SANS spectrometer at the fourth beamline of the IBR-2 reactor in the Frank Laboratory of Neutron Physics of the Joint Institute for Nuclear Research, Dubna, Russia. The wave vector *Q* was in the range from 0.007 to 0.7 Å^−1^. The wavelength was determined with the time-of-flight (TOF) method with an accuracy of about 1%. The exposure time was fixed to 30 min.

Two samples comprising alloys with 4 at.% In and 6 at.% In were studied. We will refer to these samples as G4 and G6, respectively. The measurements were carried out at 278 K, well below the solidus temperature of 288.5 K for the bulk alloy [8]. While the melting temperatures in nanostructured metals and alloys decrease with the decreasing pore size, the Ga-In alloy was found to remain solid at 278 K even within pores of 18 nm if it was preliminarily frozen [17]. We cooled down the G4 and G6 samples to 240 K to assure the complete freezing of the confined alloys. SANS measurements for the empty glass plate were also performed for comparison.

## 3. Results

**Figure 2:**
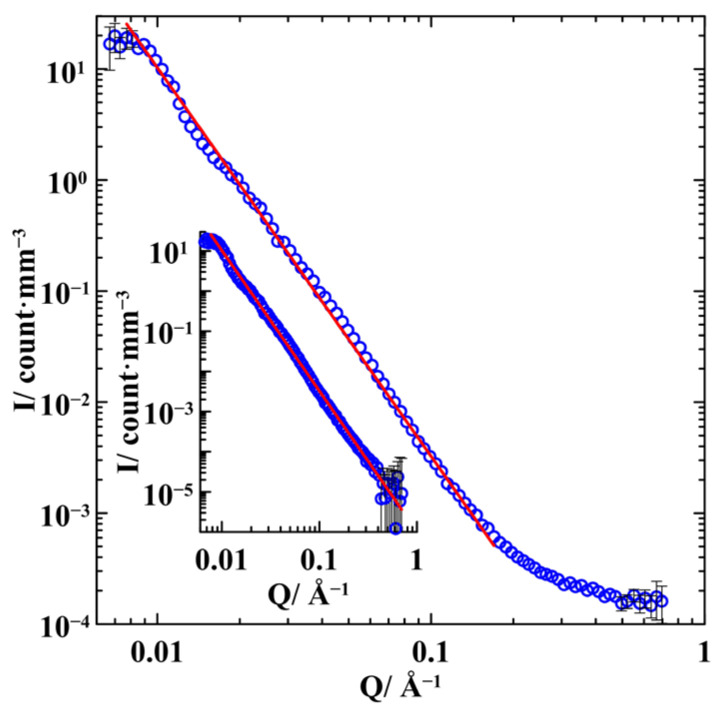
Normalized SANS intensity in the empty porous glass. The inset shows the SANS intensity after subtracting the background and incoherent scattering. The straight lines correspond to the *I*~*Q*^−3.5^ dependence.

The normalized SANS intensity for empty porous glass is shown in Figure 2. The scattering intensity *I* follows the power law *I*~*Q*^−3.5^ for wave vectors in the range from 0.008 to 0.15 Å^−1^. At higher scattering vectors, the intensity is affected by the background and incoherent scattering. The background and incoherent contributions dominate the scattering intensity above 0.5 Å^−1^ where the intensity does not depend on *Q*. These contributions can be subtracted from the experimental curve. The result is shown in the inset to Figure 2. One can see that the power law *I*~*Q*^−3.5^ extends until *Q*~0.45 Å^−1^. The deviation of the exponent from that predicted by Porod’s law indicates the fractal character of the porous glass with the surface fractal dimension *d_s_* = 2.5 in the range of scales from 1.5 to 80 nm [25]. The fractality obtained correlates with the results of Ref. [26] where the fractal dimension *d_s_* = 2.4 was found for the Vycor porous glass.

**Figure 3:**
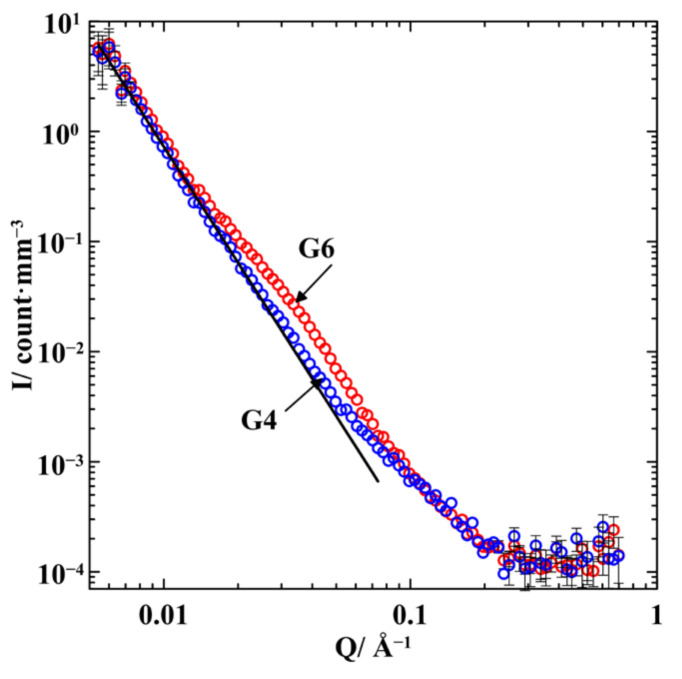
Normalized SANS intensities for G4 (empty symbols) and G6 (filled symbols). The straight line shows the *I*~*Q*^−3.5^ dependence.

The scattering intensities for G4 and G6 are shown in Figure 3. The scattering is weaker than in the empty porous glass due to the lower contrast between the silica glass and the alloy compared to the contrast between the glass and the empty pore. The scattering intensities for G4 and G6 are almost identical at *Q* < 0.014 Å^−1^ and follow the power law *I*~*Q*^−3.5^. At *Q* > 0.2 Å^−1^, the scattering is dominated by the background and incoherent contributions. The scattering intensities start diverging from the power law at much lower *Q* vectors than in the empty porous glass, revealing additional contributions of scattering by segregated alloys within pores. Figure 3 also demonstrates that scattering in G6 with higher indium composition is stronger than in G4 within the intermediate *Q* range in agreement with the scattering length densities collected in [27].

## 4. Discussion

Some rough estimates of the structure of segregates under nanoconfinement can be made using the scattering curves shown in Figure 3. The similarity of the scattering intensities for both G4 and G6 at *Q* < 0.014 Å^−1^, which follows the same power law as in the empty glass template, proves that the alloys do not contribute visibly to scattering in this range of wave vectors. Thus, we can conclude based on Bragg’s law l=2π/Q that the length scale of scatters within the confined alloys does not exceed 45 nm. The quite distinct broad hump in G6 centered near 0.04 Å^−1^ evidences the formation of segregates with sizes of the order of 10 nm. This approach to estimate the sizes of inhomogeneities was applied, for instance, to loaded opals in [28].

Note that the application of neutron scattering to the Ga-In eutectic alloy within a silica template requires strong contrasts between silica glass, gallium-rich, and indium-rich phases. The scattering length density is equal to 4.186, 3.714, and 1.559 × 10^−6^ Å^−2^ for silica glass, metallic gallium, and indium, respectively. The last two values can be also used for gallium-rich and indium-rich segregates. When using Babinet’s principle below by shifting the gallium scattering length density to zero, the contrast between gallium and the silica matrix becomes equal to 0.223 × 10^−12^ Å^−4^, while the contrast between indium and glass equals 4.644 × 10^−12^ Å^−4^. This shows the sensitivity of neutron scattering to indium segregation in the Ga-In confined nanoalloy. We should also note that indium has a large neutron absorption cross section of 4.133 cm^−1^, which limits its penetration depth. However, the indium composition in the alloy under study is below 6% and the pore volume achieves only 18% of the sample volume. As such, the neutron absorption in the loaded silica glasses remains low enough. This is in contrast to the lower penetration depths of X-rays, which require much thinner samples.

The comprehensive model of SANS of loaded porous templates should consider the contributions of scattering by the matrix and segregates and their interference. Such a model is too complicated for analysis. The problem can be simplified if we examine the difference in SANS intensities with two samples with slightly different compositions of embedded alloys. The idea of comparing the scattering curves for two or more rather similar nanocomposite samples is widely used in SANS contrast matching [29,30]. The comparison of SANS intensities in an opal matrix filled with alloys of two close compositions was also applied recently in [22].

**Figure 4:**
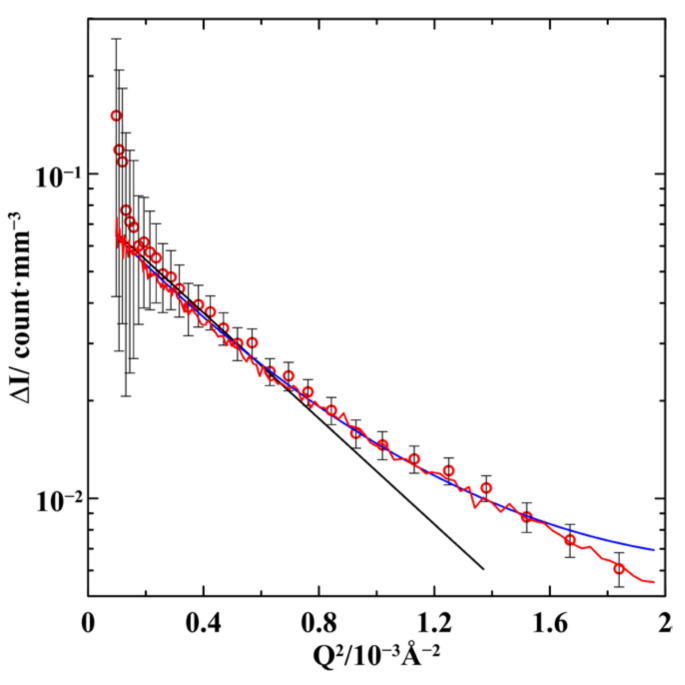
The dependence of the difference ΔI of the scattering intensities by G6 and G4 on *Q*^2^. The black line is a standard Guinier approximation, the blue line corresponds to the extended Guinier model. The red line shows the Monte Carlo simulation.

**Figure 5:**
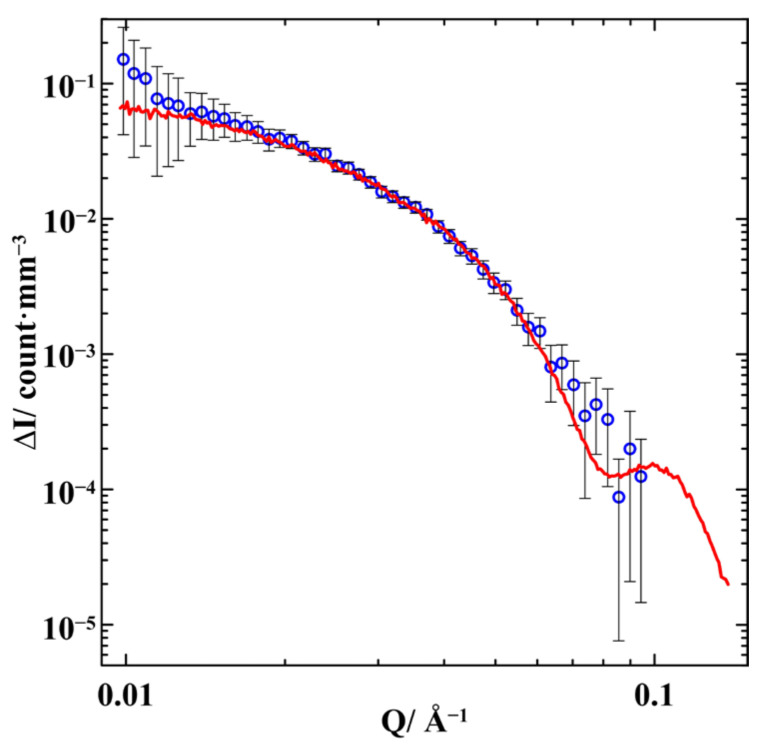
The difference ΔI(Q) of the SANS intensities by G6 and G4. The red line shows the Monte Carlo simulation for the indium-rich segregates with a shape of prolate ellipsoids of revolution with semi-axes of 17 and 5.3 nm.

Here, we discuss the treatment of the difference between the curves shown in Figure 3. We will restrict the scattering vectors to a range where the differences between the experimental data prevail over their experimental errors. The result $\Delta I=I_{G6}-I_{G4}$ of subtracting the scattering intensity for G4 from the scattering intensity for G6 is shown in Figure 4 and Figure 5 as functions of *Q*^2^ and *Q*, respectively. We start from the general relationship [23] for the scattering intensity $I_{G4}(\vec{Q})$ by the G4 composite comprising the glass matrix and two kinds of segregates enriched with gallium and with indium. The volumes of these phases are denoted as $V_{M}$, $V_{I}$, and $V_{II}$, respectively.

(1) $$I\left(\vec{Q}\right)={\left|\int_{V_{M}}\rho_{M}\left(\vec{r}\right)e^{i\vec{Q}\vec{r}}d\vec{r}+\int_{V_{I}}\rho_{I}e^{i\vec{Q}\vec{r}}d\vec{r}+\int_{V_{II}}\rho_{II}e^{i\vec{Q}\vec{r}}d\vec{r}\right|}^{2}.$$

In (1), $\rho_{M}$, $\rho_{I}$, and $\rho_{II}$ are scattering length densities of the glass matrix and gallium- and indium-rich segregates. By also using Equation (1) for the G6 sample, and following Ref. [22], we can obtain the difference $\Delta I(\vec{Q})$:

(2) ΔIQ→=ρII−ρI2∫ΔVeiQ→r→dr→2+2Re∫ΔVeiQ→r→dr→⋅∫VIIeiQ→r→dr→¯,

where ΔV is a difference in volumes occupied by the indium-rich phase in G6 and G4. To obtain Equation (2), Babinet’s principle [23] was applied to both G4 and G6 nanocomposites, and the scattering length densities of the glass matrix and segregates were decreased by $\rho_{I}$. The conversion of Equation (1) into Equation (2) implies that the matrices of G4 and G6 are similar, which is true, on average, for large enough samples. We also assumed, following Ref. [22], that the small additional volume ΔV of the indium-rich phase in G6 was related to an increase in the number of the indium-rich scatters or in their sizes.

The second term in Equation (2) accounts for the interference of scattering by the main and additional indium-rich segregates with the $V_{II}$ and ΔV volumes, respectively. Within the framework of the model of independent scatters, the second term in Equation (2) can be neglected. This simplified model can be described using the Guinier method, which allows finding the gyration radius of scatters without suppositions about the scatter shape [23]. The Guinier method predicts that the SANS intensity by scatters with a gyration radius *R_g_* is given by:

(3) $$\Delta I(\vec{Q})=Ce^{-\frac{R_{g}^{2}}{3}Q^{2}},$$

where *C* is a numerical fitting coefficient. The result of the fit is shown in Figure 4. The most probable gyration radius *R_g_* was found to equal *R_g_* = 7.5 nm. As the Guinier method is based on the Maclaurin expansion, it is justified only for *Q* < 1.3/*R_g_*. This upper boundary corresponds to *Q* ≃ 0.017 Å^−1^. To extend the limits of the Guinier method, it was suggested in [24] to use more terms of Maclaurin series. The extended Guinier approximation is

(4) $$\Delta I(\vec{Q})=Ce^{-\frac{R_{g}^{2}}{3}Q^{2}+\frac{3M_{4}-R_{g}^{4}}{180}Q^{4}}.$$

here *M*_4_ is the radial fourth moment of the scatter

(5) $$M_{4}=\frac{1}{V_{s}}\int_{V_{s}}r^{4}d\vec{r},$$

where $V_{s}$ is a scatter volume. The extended Guinier approximation is justified for *Q* < 3/*R_g_*. The fitting curve obtained with the extended Guinier approximation is also shown in Figure 4. The most probable values of the gyration radius and fourth moment are *R_g_* = 8 nm and $M_{4}^{1/4}$ = 8 nm. The upper boundary of the extended Guinier method is equal to *Q* ≃ 0.04 Å^−1^. As can be seen in Figure 4, the extended Guinier approximation provides quite a good fit for the experimental data up to the upper boundary of its validity. The standard Guinier curve starts deviating from the experimental data near *Q*~0.02 Å^−1^, also close to the upper limit of the model.

The more accurate analysis for a larger range of scattering vectors should be based on Equation (2). The calculations were performed using the Monte Carlo method for the volume of 10 × 10 × 10 µm and averaged over 1000 iterations. We considered various shapes of the indium-rich segregates: spheres, ellipsoids, and cylinders. For every shape, we changed the segregate sizes. The segregates could be located randomly within the template pores or their positions could be correlated. The increase in the volume of the indium-rich segregates could be related to the increase in the sizes of segregates or in their number. The total amount of the segregates matched with the alloy composition constraints.

The best fit for the difference $\Delta I(\vec{Q})$ in the SANS intensities is shown in Figure 4 and Figure 5. It was obtained under the assumption that the indium-rich segregates are prolate ellipsoids of revolution with semi-axes of 17 and 5.3 nm. The ellipsoids were randomly located within pores. When the indium composition in the Ga-In alloy increased in G6 compared to G4, the number of the ellipsoid segregates also increased. Other assumptions about geometric characteristics of segregates led to a much worse agreement with the experimental SANS data. To compare the results of the Monte Carlo calculations with those of the Guinier approaches, we evaluated the gyration radius and radial fourth moment: *R_g_* = 8.3 nm and $M_{4}^{1/4}$ = 9.5 nm. There is an excellent agreement between the pictures of the confined alloy structure obtained by the computer simulation method based on the initial formulae for neutron scattering and the common Guinier and extended Guinier models. The estimates of the segregate sizes also do not contradict the ordinary estimates based on the scattering hump position in the G6 sample.

The calculated curve shown in Figure 5 was obtained for an assumption of monodisperse ellipsoid segregates. The fact that the agreement with experimental data is perfect within experimental accuracy denies the presence of noticeable numbers of scatters with significantly different sizes. However, small alterations in the size of segregates (weak polydispersity) do not move the calculated curve beyond the error bars. Therefore, we should consider a model of prolate ellipsoid scatters with specific axes as an averaged picture.

Note that the sizes and shapes of indium-rich segregates found in the present work differ remarkably from those obtained for the Ga-In alloy of the same compositions embedded into opal templates [22]. This suggests the pronounced influence of pore geometry on segregation in confined nanoalloys.

**Figure 6:**
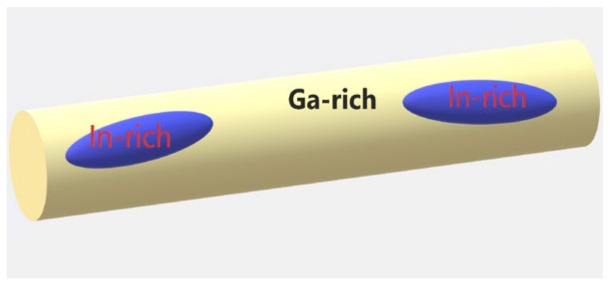
The pore segment loaded with gallium- and indium-rich phases.

We now discuss the compatibility of the results obtained by the Monte Carlo calculations and crystallization process in the confined nanoalloys. As the indium composition in both alloys is less than the eutectic 14 at.% amount [8], the crystallization in the melt starts upon cooling with precipitation of the gallium-rich segregates. These segregates grow gradually with further cooling until the composition of the residual melt equals the eutectic one. Just above the solidus line, near 71 at.% and 57 at.%, gallium became frozen in the G4 and G6 samples, respectively. We can assume according to the numerical calculations that the unfrozen melt of the eutectic composition occupies parts of pore segments of about 34 nm in length; then, the melt segregates with precipitation of the indium-rich phase. It is plausible that precipitation of gallium begins near pore walls, which can serve as crystallization centers. Therefore, the indium-rich segregates are supposed not to touch the pore walls and are likely positioned closer to the pore middle as shown in the cartoon in Figure 6. Because of low indium composition, we could assume that the confined indium-rich segregates do not interact with each other. However, this assumption is not crucial and was not used in the Monte Carlo simulation since the interference of neutrons scattered by different segregates was taken into account.

## 5. Conclusions

In this study, we applied the recently suggested simulation method based on the initial formulae for neutron scattering [22] to the analysis of the structure of the frozen eutectic gallium-indium alloys embedded into a nanoporous silica glass template. We showed that some information about the geometry of the segregates can be obtained from SANS intensities for two nanocomposites with confined alloys of slightly different compositions. While the structure of the alloys cannot be reconstructed unambiguously using only the SANS measurements, the results of the Monte Carlo simulation appeared to be consistent with the estimates made by the standard and extended Guinier approximations. The results also agreed qualitatively with the scattering hump position. In the particular case considered in the present work, the numerical calculations predicted a shape of the prolate ellipsoid of revolution with semi-axes of 17 and 5.3 nm for the indium-rich segregates, which were randomly located within nanopores.

## Data Availability

Not applicable.
